# The Effect of Probiotic Supplements on Metabolic Parameters of People with Type 2 Diabetes in Greece—A Randomized, Double-Blind, Placebo-Controlled Study

**DOI:** 10.3390/nu15214663

**Published:** 2023-11-03

**Authors:** Eva Zikou, Nikolas Dovrolis, Charilaos Dimosthenopoulos, Maria Gazouli, Konstantinos Makrilakis

**Affiliations:** 1 First Department of Propaedeutic Internal Medicine, National and Kapodistrian University of Athens Medical School, Laiko General Hospital, 11527 Athens, Greece; zikoudiatrofi@gmail.com (E.Z.); harisdimos@gmail.com (C.D.); 2 Department of Basic Medical Sciences, Laboratory of Biology, National and Kapodistrian University of Athens Medical School, 11527 Athens, Greece; ndovroli@med.duth.gr (N.D.); mgazouli@med.uoa.gr (M.G.)

**Keywords:** probiotics, diabetes type 2, gut microbiome

## Abstract

The role of probiotic supplementation in type 2 diabetes (T2D) treatment is controversial. The present study aimed to assess the effects of a multi-strain probiotic supplement (LactoLevure^R^ (containing *Lactobacillus acidophilus*, *Lactobacillus plantarum*, *Bifidobacterium lactis*, and *Saccharomyces boulardii*)) over 6 months, primarily on glycemic control as well as on lipid levels and alterations in the gut microbiome, among individuals with T2D residing in Greece. A total of 91 adults with T2D (mean age [±SD] 65.12 ± 10.92 years, 62.6% males) were randomized to receive the probiotic supplement or a matching placebo capsule, once daily, for 6 months. Blood chemistries and anthropometric parameters were conducted every 3 months, and stool samples were collected at baseline and at 6 months. Significant reductions in HbA1c, fasting blood glucose, and total cholesterol were observed in participants treated with the probiotic supplement (n = 46) compared to the controls (n = 45), even after adjustment for a greater decrease in adiposity (waist circumference). Although there were no statistically significant differences in the diversity of the gut microbiome (α and β diversity), the administration of probiotics did influence several genera, metabolites, and key enzymes associated with diabetes. Overall, the administration of the multi-strain probiotic LactoLevure^R^ over a 6-month period in individuals with T2D was well-tolerated and had a positive impact on metabolic parameters, alongside improvements in indices of adiposity.

## 1. Introduction

Type 2 diabetes (T2D) is a common, chronic metabolic disease, with an increasing prevalence often linked to rising obesity rates and an aging population [1]. Its etiology has traditionally been attributed to a blend of genetic and environmental factors, particularly excessive caloric intake and sedentary lifestyles [2]. Although it is primarily characterized by disturbances in both insulin secretion and action, its pathogenesis is multifaceted and can vary significantly among individuals [3]. This complexity can lead to various associated health issues, including inflammation, dyslipidemia, hypertension, and a range of metabolic abnormalities, ultimately resulting in serious complications. Despite the availability of numerous pharmaceutical treatments [4], the management of T2D remains suboptimal. Typically, blood glucose levels progressively increase over time in most T2D patients [5], necessitating intensified treatment to address evolving pathophysiological changes. Consequently, there is a growing interest in developing cost-effective healthcare products aimed at more effectively regulating blood glucose levels.

Recent years have witnessed a surge in interest in the gut microbiome—the astonishingly vast and diverse community of bacteria, fungi, and viruses residing in and on the intestines—as a contributing factor in the pathogenesis of various diseases [6], among them obesity [7], metabolic syndrome, and diabetes mellitus [8]. It has been postulated that the composition and functionality of the gut microbiome can affect a range of pathways, including those related to immunity, energy, lipid, and glucose metabolism [9]. The gut microbiome is intimately linked to the immune system, as it helps train and modulate immune response by interacting with immune cells in the gut-associated lymphoid tissue (GALT). It thus helps maintain the balance between pro-inflammatory and anti-inflammatory responses, contributing to overall immune system homeostasis [10]. It is also involved in the fermentation of dietary fibers and the production of short-chain fatty acids (SCFAs), such as acetate, propionate, and butyrate, which serve as energy sources for the host and play a role in regulating appetite and fat storage, ultimately impacting energy metabolism [11]. The gut microbiome can also influence lipid metabolism by modulating the absorption of dietary lipids, promoting the conversion of cholesterol into bile acids, and affecting the expression of genes involved in lipid metabolism in the liver [12]. Emerging evidence suggests that the gut microbiome plays a crucial role in glucose metabolism and insulin sensitivity as well [13]. Abundance and composition differences in the intestinal microbiome are likely associated with the development of T2D, with diabetic patients exhibiting distinct microbiome profiles compared to healthy individuals, often marked by reduced populations of beneficial butyrate-producing bacteria and an increase in various pathogens [14]. Within this complex microbial community, *Firmicutes* and *Bacteroides* emerge as the predominant phyla responsible for butyrate production [15]. Notably, these microbial taxa are closely associated with potential benefits in the management of T2D, primarily through their influence on glucose regulation [16].

Probiotics, defined as living microorganisms with health benefits to humans when administered in adequate amounts [17], have been extensively studied for their potential to address various disorders. Of note, according to the Food and Agriculture Organization (FAO) of the United Nations and the World Health Organization (FAO/WHO), the proof of viability of live or active cultures at a minimum level, reflective of typical levels seen in fermented foods, has been suggested to be 1 × 10^9^ colony forming units (CFU) per serving [18]. While *lactobacilli* and *bifidobacteria* strains are the most employed probiotics, other microorganisms, including the yeast *Saccharomyces boulardii*, have also been employed. Numerous animal studies focusing on diabetes models have highlighted the positive effects of specific probiotic bacterial strains on glucose regulation [19,20]. However, human studies on diabetes have yielded conflicting results, with most being of short duration (typically less than three months) or marked by heterogeneous outcome reporting, often concentrating on fasting blood glucose (FBG) rather than HbA1c [21,22,23,24,25]. Furthermore, the relationship between probiotic administration and the gut microbiome has not been consistently explored. Consequently, while there is evidence suggesting a connection between the gut microbiome and the risk of adiposity-related comorbidities such as T2D, a causal link has not been established [26], and there is no clear consensus in the literature that modulating the gut microbiome—via probiotics or other means—effectively treats obesity and T2D [7]. Since probiotic supplements are not standardized and the efficacy can vary widely between different products and strains, it can be challenging to determine which probiotics are most effective for glycemic—and generally metabolic—control. Numerous studies have explored the effectiveness of different probiotic strains and their combinations in the context of treating T2D. Notably, probiotic yoghurt containing *Lactobacillus acidophilus* and *Bifidobacterium lactis* has shown promise in improving the lipid profile of T2D patients [27]. *Lactobacillus acidophilus* has demonstrated its potential for anti-diabetic effects by inducing favorable changes in the epithelial barrier function, ultimately leading to a reduction in inflammation. Additionally, it plays a role in regulating genes that impact glucose and lipid metabolism [28]. Similarly, *Lactobacillus plantarum* has emerged as a promising candidate for the management of T2D. This strain is known for its involvement in regulating glucose metabolism within the liver, contributing to the restoration of gut microbiota composition and actively participating in the reduction of low-grade inflammation. While there have not been any human trials involving *Lactobacillus plantarum* to date, animal model investigations have demonstrated its potential in mitigating hyperglycemia and insulin resistance [29,30,31,32]. Furthermore, studies with *Saccharomyces boulardii,* although primarily conducted in animal models, have also shown promising results [33,34,35]. Therefore, investigating the combined effects of these probiotic strains in T2D patients seems to be a logical next step in assessing their efficacy. To that effect, specific individual products in the market must be tested. Moreover, since the gut microbiome has been shown to differ according to geography [36], representing a significant confounding factor in studies examining the effects of population-specific diets and lifestyles, investigating region-specific alterations in the gut microbiome and their metabolic implications is essential. To date, there has been no such study conducted in Greece, and this dearth of local data of probiotic-induced modifications of the gut microbiome in persons with diabetes was an incentive to conduct this study. 

Therefore, the aim of the present study was to conduct a randomized, double-blind, placebo-controlled trial over a sufficient duration (six months) to evaluate the impact of probiotic supplementation primarily on glycemic control (HbA1c), as well as lipid levels, obesity parameters, and associated changes in the gut microbiome in individuals with type 2 diabetes in Greece.

## 2. Materials and Methods

### 2.1. Participants

The study enrolled adult individuals diagnosed with type 2 diabetes (T2D) who received care at the outpatient Diabetes Center of Laiko General Hospital in Athens, Greece. Participants met the study’s inclusion and exclusion criteria and willingly agreed to take part. 

The inclusion criteria were as follows:

(a) A diagnosis of T2D (based on the American Diabetes Association criteria [37]), for a duration exceeding 6 months.
(b) Age above 18 years.
(c) A body-mass index (BMI) greater than 18.5 kg/m^2^.
(d) HbA1c levels exceeding 6%.
(e) Stable use of antidiabetic medications (oral or injectable GLP-1 RAs) for at least 8 weeks before screening.

The exclusion criteria were as follows:

(a) Type 1 diabetes or other forms of diabetes.
(b) Pregnancy or plans to become pregnant during the study.
(c) End-stage kidney failure requiring dialysis, or presence of other severe diseases such as cancer or significant hepatic insufficiency (AST/ALT levels more than 3.5 times above normal).
(d) Recent use of other probiotic products or antibiotics within the previous 6 months.
(e) Participation in other ongoing clinical trials.
(f) Presence of any other medical condition that, in the opinion of the investigators, could hinder compliance with the study protocol, such as malabsorption syndrome (for example celiac disease, inflammatory bowel disease, chronic pancreatitis, cystic fibrosis, short bowel syndrome, bacterial overgrowth, parasitic infections, bariatric surgery, autoimmune disorders like systemic lupus erythematosus and systemic sclerosis, etc.), or an inability to take orally administered medications (due, for example, to dysphagia, Parkinson’s, Alzheimer’s and motor neuron diseases, throat or esophageal problems, stroke, and other neurological conditions).
(g) Administration of insulin. The decision to exclude patients on insulin was based on previous studies showing a more substantial reduction in HbA1c with probiotics in participants not receiving insulin compared to those on insulin therapy [38].

Sample size calculation was created considering 80% power at α = 0.05 to detect a decrease in HbA1c of at least 0.3 percentage points. Based on this, we calculated that we would need to recruit at least 88 individuals for the study. All participants were given verbal and written information about the study and signed an informed consent form, according to the recommendations of the Declaration of Helsinki [39], prior to their enrollment in the study.

### 2.2. Study Design

This study was a single-center, double-blind, placebo-controlled, randomized clinical trial, with a 6-month duration, involving the administration of probiotic supplements to persons with T2D. Ethical approval was obtained from the Institution Review Board of Laiko General Hospital in Athens, Greece (approval number 306/26.04.2021). The trial protocol was registered on clinicaltrials.gov (identifier NCT06032988). 

Eligible participants were randomized using a computer generator (www.randomization.com, accessed on 27 April 2021), which randomized each subject to a single treatment by using the method of randomly permuted blocks, to receive either a multi-strain probiotic supplement (in the form of a capsule) or a matching placebo capsule, both taken once daily. The probiotic capsule (LactoLevure^R^) contained the following bacterial strains as per the manufacturer’s information: *Lactobacillus acidophilus* (1.75 × 10^9^ colony forming units (CFU)), *Lactobacillus plantarum* (0.5 × 10^9^ CFU), *Bifidobacterium lactis* (1.75 × 10^9^ CFU), and *Saccharomyces boulardii* (1.5 × 10^9^ CFU). These capsules, both the probiotic (LactoLevure^R^) and the placebo, were prepared and provided to the study personnel through an unrestricted research grant from Uni-Pharma Greece (Uni-Pharma Pharmaceutical Laboratories S.A., Kifisia, Greece), which had no further involvement in the study’s planning, execution, or data analysis. The probiotic and placebo capsules were indistinguishable in appearance and packaging, ensuring that neither the participants nor the investigators were aware of the treatment assignments in this double-blind study. Additionally, participants were instructed to maintain their existing dietary and exercise habits and refrain from consuming yoghurt or similar dietary supplements during the study (this was reminded to them and verified at each subsequent visit). Care was taken to keep other pharmaceutical medications unchanged throughout the study; participants who needed to initiate insulin treatment or modify their oral antidiabetic medication doses were excluded from further follow-up and analysis.

Participants were followed at the outpatient Diabetes Center every 3 months, aligning with standard practice for individuals with diabetes. At the baseline visit, and subsequently at the 3-month and 6-month intervals, anthropometric measurements were recorded, including height (in cm), weight (in kg), blood pressure (in mmHg), and waist circumference (in cm), and body mass index (BMI, kg/m^2^) was calculated. Weight and height were measured with the participants minimally clothed, without shoes, in a standing position. Waist circumference was measured with the help of an inelastic tape measure, at the mid-point between the iliac crest and the costal arch, as per the WHO guidelines [40]. Blood pressure was measured twice, 2 min apart, in a sitting position, after 10 min of rest, and the average of the two was recorded. An automated accredited [41] Omron electronic sphygmomanometer (Omron HBP T105) was used for the measurements. All these measurements were consistently performed by the same trained personnel at all stages of the study.

Additionally, blood biochemical parameters, including glycated hemoglobin (HbA1c), fasting blood glucose (FBG), fasting lipid panel (total cholesterol, triglycerides, HDL-cholesterol, and LDL-cholesterol), renal function indicators (blood urea and serum creatinine), and liver function tests (AST, ALT, γGT, and alkaline phosphatase), were extracted manually from participants’ medical records at the baseline, 3-month, and 6-month visits.

Furthermore, participants were asked to give a stool sample at the beginning and the end (6 months) of the study for gut microbiome analysis. They were provided with instructions to collect samples at home in the morning. These samples were then transported to the clinic under refrigeration and stored in a freezer (−20 °C) until processing. Researchers responsible for the procedure utilized the Fecal Swab Collection and Preservation System/Stool DNA Isolation Kit provided to them. The collected samples were stored in the freezer until the entire collection process was completed at the follow-up visit of 6 months. This DNA isolation was analyzed through 16S rRNA Sequencing, and a microbial analysis through alpha and beta diversity was held for any quantitative or qualitative alterations, respectively [42]. Additionally, the Tax4Fun2 pipeline contributed to the identification of enzymes and metabolic pathways [43]. 

The primary outcome of this study was the change in HbA1c between the two groups (active probiotic treatment and control) at 6 months. Anthropometrics (especially obesity parameters), blood lipid levels, liver function tests, and gut microbiome changes were measured as secondary outcomes. A questionnaire was also administered at the last visit (6 months) to collect data on participants’ tolerance and satisfaction with the treatment, including the frequency of symptoms such as constipation, diarrhea, bowel function, bloating, gas production, and abdominal pain. 

#### 16S rRNA Sequencing

External and independent facilities (MR DNA, Molecular Research LP, Shallowater, TX, USA) were contracted to provide sequencing services for the study. The sequencing process was carried out using the Illumina MiSeq platform, a high-throughput next-generation sequencing system, following the manufacturer’s guidelines at MR DNA’s website (www.mrdnalab.com (accessed on 14 November 2022), Shallowater, TX, USA). To ensure high quality, the sequenced reads were subjected to rigorous quality control measures, where any sequences below 150 base pairs and those with ambiguous base calls were removed. After dereplication, the unique sequences were denoised and had chimeras removed, resulting in a denoised sequence or zero-radius operational taxonomic unit (zOTU). These zOTUs represent a group of unique sequences that are clustered based on their sequence similarity. To determine the taxonomic identity of the zOTUs, BLASTn, a sequence similarity search tool, was utilized against a curated database obtained from the National Center for Biotechnology Information (NCBI). A 99% sequence similarity threshold was used to ensure high accuracy of taxonomic classification. The final library contained samples that were aligned and matched to the zOTUs, resulting in an average of 28,330 aligned reads per sample. The zOTU-based approach is advantageous because it allows for the identification of microbial taxa at a high resolution, providing a more accurate representation of microbial diversity.

### 2.3. Bioinformatics and Statistical Analysis

Categorical variables are reported as frequencies and relative frequencies (%) and continuous quantitative variables as mean ± standard deviation. The normal distribution of variables was tested with the Shapiro–Wilk test. For comparisons between categorical variables, the Χ^2^ test was used, and for comparisons between continuous variables, the Student’s t-test for independent samples was used (for normally distributed variables) or the non-parametric Mann–Whitney U test for non-normally distributed variables. Pearson’s correlation coefficient (r) or Spearman’s Rho coefficient (for non-normal distributions) were used for the evaluation of statistical correlations between variables. To comprehensively examine the effects of time, group, and their interactions, a two-factor mixed repeated-measure ANOVA was employed. This analysis incorporated the independent variables of “time”, which had three levels (baseline, 3 months, and 6 months), and “group”, which had two levels (intervention and control). It is worth noting that the assumption of sphericity was not met, necessitating appropriate adjustments. Clinical and biochemical parameters were treated as dependent variables. To account for multiple comparisons between different time points, the Bonferroni correction was applied to control the *p*-value. The statistical package Statistical Package for Social Sciences (SPSS, Inc., Chicago, IL, USA) version 28.0 was used for the analyses. The significance of the tests was determined as *p* < 0.05.

To analyze the stool data collected during the research, several steps were undertaken. Initially, the raw read counts, sample metadata, and taxonomy information files were meticulously formatted and utilized as input data within the MicrobiomeAnalyst platform [44]. To ensure the statistical robustness of the analysis, the initial 1319 zOTUs were filtered down to 452 after removing 816 low-abundance (<20% prevalence in all samples) and 51 low-variance (<10% based on the inter-quantile range) features. To account for any isolation/sequencing biases, samples were normalized using total sum scaling (TSS). The samples were sub-grouped based on receiving probiotics or placebo as well as the time of sampling (0 and 6 months timepoints). These groupings were: (i) all patients at timepoint 0 that would receive the placebo (Group A1); (ii) all patients at timepoint 0 that would receive the probiotic (Group B1); (iii) all patients at 6 months who had received the placebo (Group A2); and (iv) all patients at 6 months who had received the probiotic (Group B2). Alpha diversity analysis was carried out on the zOTU level using the Shannon index on unfiltered raw counts. For beta diversity, NMDS and ANOSIM were utilized, and for univariate differential abundance calculations, DESEQ2 was employed at the genera and species levels, considering fold change (FC ≥ 2) and *p*-values (*p* < 0.05). Additionally, functional prediction of the microbial communities was performed using Tax4Fun2 [43] on the KO (KEGG Ortholog) level [45]. KOs were mapped to the corresponding metabolites using a custom API-call script to the KEGG-API. Statistical analysis between the predicted microbial metabolites and pathways was performed using the standalone application STAMP [46], using a two-sided Welch’s *t*-test.

## 3. Results

**Figure 1:**
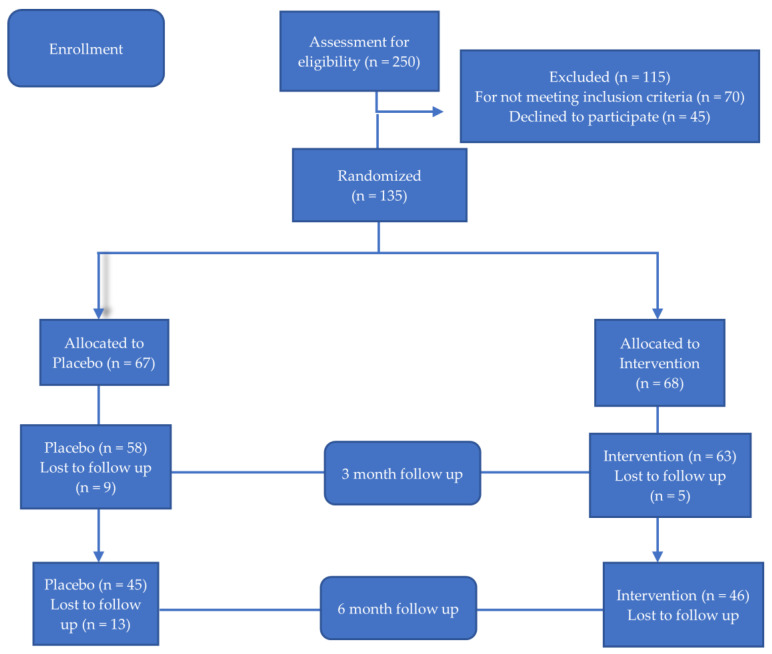
Flow chart detailing participants’ recruitment, randomization, and allocation.

A total of 250 people with T2D were screened for eligibility based on the chosen inclusion/exclusion criteria, as illustrated in Figure 1. Of these, 115 were deemed ineligible and subsequently excluded from the study. The final cohort comprised 135 individuals (age [mean ± SD] 65.1 ± 10.9 years, BMI 32.7 ± 6.9 kg/m^2^, 62.6% males), who were randomly allocated to receive the probiotic (n = 68) or placebo (n = 67) in a double-blind manner, for 6 months, in parallel to their nutrition and medication treatment. Out of these 135 participants, 91 successfully completed the 6-month protocol (46 in the intervention group and 45 in the control). The drop-outs were comparable between the groups and concerned 44 persons (22 in each group) for the following reasons: (a) one participant, from the placebo group, experienced a fatality; (b) three individuals (two from the intervention group and one from the placebo group) faced difficulties with swallowing; (c) seven participants (three from the intervention group and four from the placebo group) reported fatigue from the 6-month supplement regimen; (d) two participants (one from each group) required emergency surgery; (e) nine participants (six from the intervention group and three from the placebo group) perceived no benefit from the intervention and decided to discontinue; (f) six participants (two from the intervention group and four from the placebo group) unintentionally missed doses; (g) eight participants (two from the intervention group and six from the placebo group) needed to transition to insulin treatment or modify their oral antidiabetic medication doses; and (h) eight participants (five from the intervention group and three from the placebo group) opted to terminate their follow-up. Importantly, individuals who withdrew from the study did not display significant differences in age or anthropometric and clinical characteristics when compared to those who remained in the study.

Within this cohort, 29 participants, initially randomized from the larger pool, consented to provide stool samples, thereby participating in a microbiome sub-study (15 in the intervention group and 14 in the control group). Of these, 23 successfully completed the 6-month protocol (12 in the intervention group and 11 in the control group) and were included in the microbiome sub-study, as depicted in Supplementary Figure S1. 

Table 1 shows the baseline demographic, clinical, and laboratory characteristics of the intervention and control groups (n = 91). The allocation of participants in the two groups was balanced, and there were no differences in the examined parameters. Regarding antidiabetic medical treatment, a similar number of participants in the two groups were on metformin treatment (31 in the intervention and 30 in the placebo group) as well as on glucagon-like peptide-1 receptor agonist (GLP-1 RA) therapy (32 in the intervention and 27 in the placebo group) (all *p* non-significant).

**Figure 2:**
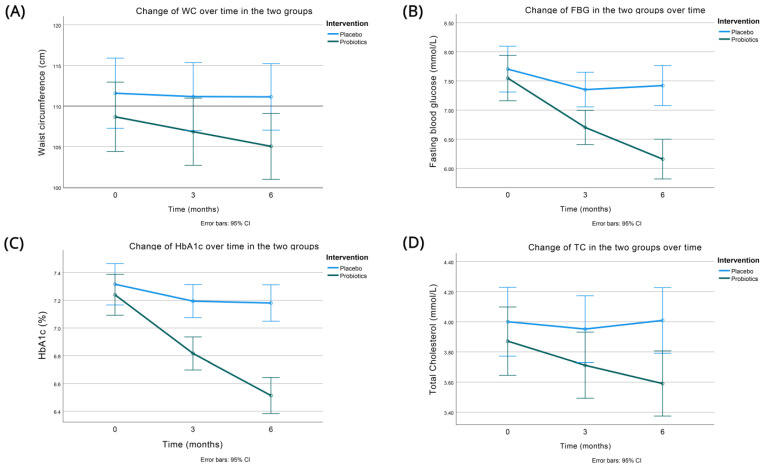
Changes in waist circumference (**A**), fasting blood glucose (**B**), HbA1c (**C**), and total cholesterol (**D**) between the two groups over time (baseline, 3, and 6 months of intervention).

Table 2 shows the effects of the intervention on anthropometric characteristics in the two groups at the 3- and 6-month follow-up visits. As depicted, there was a significant decrease in adiposity, as measured by the waist circumference (Figure 2A), favoring the intervention group (it decreased by −3.63 (3.10) vs. −0.44 (5.44) cm in the probiotic and placebo group, respectively (*p* < 0.001)). BMI showed a trend for improvement more in the probiotic group, but the difference did not reach statistical significance (*p* = 0.085). Systolic and diastolic blood pressures were not appreciably affected.

Moreover, there were significant improvements in the glycemic and lipid parameters at both the 3- and 6-month follow-up evaluations, with these benefits being more pronounced in the intervention group (Table 3 and Figure 2B–D).

Specifically, the primary study outcome, HbA1c, experienced a significant reduction, with a noteworthy difference of 0.59 percentage points (%) in the decrease of HbA1c between the groups (−0.73 (0.42) vs. −0.14 (0.46) % for the intervention and placebo groups, respectively) at the 6-month follow-up (*p* < 0.001), even after adjustment for the decrease in waist circumference. Of note, a change of >0.5% in the HbA1c is generally considered clinically significant [47]. A reduction in HbA1c was particularly pronounced in participants with a baseline HbA1c > 7% (47.3% of participants), with a between-group HbA1c difference of 0.64%, compared to their counterparts with baseline HbA1c ≤ 7% (HbA1c difference between the groups of 0.61%).

For fasting blood glucose (FBG), the respective reduction at 6 months amounted to a difference of 1.11 mmol/L (−1.39 (1.08) vs. −0.28 (0.96) mmol/L for the probiotics and placebo groups, respectively), once again favoring the intervention group (*p* < 0.001). The positive effect of probiotics on FBG was more marked in participants with less controlled glycemia at baseline (baseline FBG > 7.22 mmol/L, affecting 47.3% of participants), with an FBG difference between the groups of 1.41 mmol/L, compared to those with a baseline FBG ≤ 7.22 mmol/L, which displayed an FBG difference of 0.77 mmol/L.

Total cholesterol also showed significant improvements during the 6 months of intervention, with a difference of 0.29 mmol/L (−0.28 (0.27) vs. 0.01 (0.50) mmol/L), favoring the probiotic group (*p* = 0.012). Of note, all these differences (on glycemic indices and lipids) remained significant even after controlling for the greater decrease of adiposity in the probiotics group (measured as a decrease in waist circumference). 

There were favorable trends in the other lipid parameters (decrease in triglycerides, increase in HDL-cholesterol, and decrease in LDL-cholesterol), favoring the probiotic group, although these differences did not reach statistical significance. 

Furthermore, no substantial differences in liver enzyme levels were detected between the two groups during the intervention. 

All participants, in both the control and intervention groups, were surveyed at their last visit regarding their experience with probiotic intake, specifically in relation to constipation, diarrhea, bowel function, bloating, gas production, and abdominal pain. In the control group, the majority (71%) reported no discernible differences, while 9% noted a minor improvement, and 20% perceived a significant improvement in bowel function. In contrast, among the intervention group, 30% reported no changes, 35% experienced a minor improvement, and 35% noted a significant improvement (*p* < 0.001). 

### Microbiome Sub-Study

In the microbiome sub-study, participants’ baseline characteristics were balanced between the intervention and the placebo groups, except for the waist circumference, which was higher in the control group compared to the active treatment arm (Supplementary Table S1). A similar number of participants in the two groups were on metformin treatment (9 in the intervention and 8 in placebo), as well as on GLP-1 RA therapy (10 in the intervention and 6 in the placebo group). A change in the investigated clinical and biochemical parameters showed a similar pattern as in the whole group of participants. Adiposity indices (BMI and WC) decreased significantly more in the probiotic group compared to the placebo (Supplementary Table S2), and significant decreases were also observed in the glycemic indices (HbA1c and FBG), as well as the lipid parameters (triglycerides), in favor of the intervention group, even after adjustment for the adiposity changes (Supplementary Table S3). Liver function parameters were not affected. 

**Figure 3:**
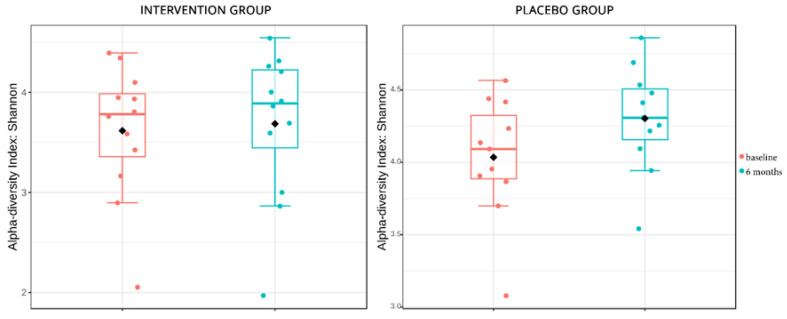
α diversity metrics in the control and intervention group, used to identify the stability of the microbial ecology and changes in the composition affected by the intervention. Diamond = mean; Horizontal Line = median.

**Figure 4:**
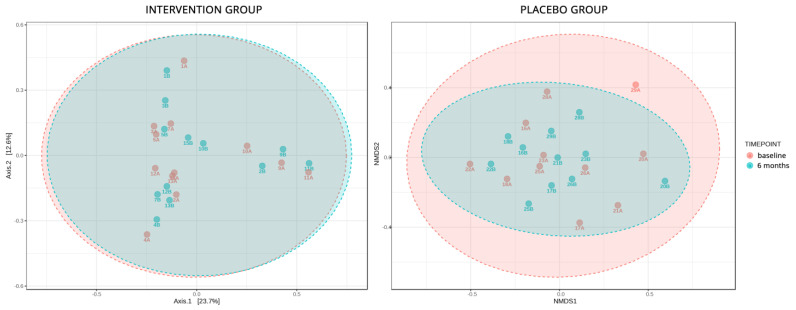
Analysis of the qualitative change in species and genera of bacteria in the control and intervention group.

The stool analyses employed in this sub-study aimed to identify the differences in microbial composition mediated by the disease and the intervention. To identify the stability of the microbial ecology and changes in the composition affected by the intervention, α diversity metrics on Group A2 versus Group A1 and Group B2 versus Group B1 were applied. Both analyses showed a non-significant uptick in α diversity (*p* = 0.1 and *p* = 0.8 respectively), which signifies the changes brought by the passing of time, but the B groups appeared to be a bit more stable in biodiversity even though probiotics were introduced to their diet (Figure 3). The same non-significant changes were also depicted in the β diversity analyses, in which, qualitatively, the microbial composition had not changed significantly in all patients, except in one from the placebo group (Figure 4). When comparing statistical changes in individual microbial taxa at baseline and after 6 months, changes were detected in both the placebo and the intervention group. For the former, the genera *Coprobacillus*, *Klebsiella*, *Collinsella*, *Lachnobacterium*, *Shigella*, *Escherichia*, *Parasporobacterium*, *Caloramator*, and *Ruminococcus* were significantly diminished, while *Sutterella* and *Haemophilus* were enriched. As for the intervention group, the genera *Akkermansia*, *Megamonas*, *Flavonifractor*, and *Shigella* showed a significant decrease in their relative abundance, while *Lachnospira* and *Pantoea* increased their populations.

**Figure 5:**
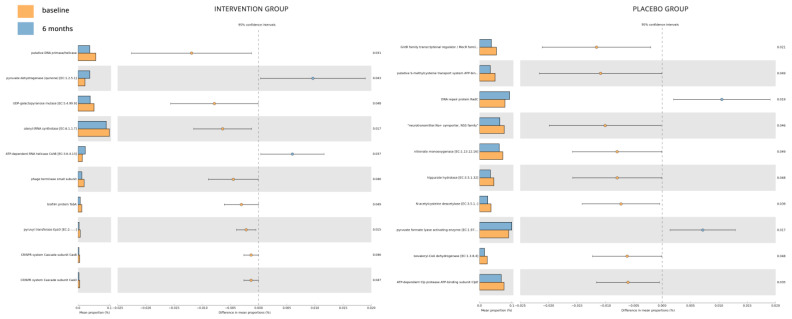
Tax4Fun2 pipeline, used to predict functional changes based on the differential abundance of the sample groups.

The Tax4Fun2 pipeline allowed for the prediction of functional changes based on the differential abundance of the sample groups (Figure 5). The top 10, by effect-size, perturbed KOs for the placebo group highlighted a decrease of “GntR family transcriptional regulator/MocR family aminotransferase”, “putative S-methylcysteine transport system ATP-binding protein”, “neurotransmitter: Na+ symporter, NSS family”, “nitronate monooxygenase [EC:1.13.12.16]”, “hippurate hydrolase [EC:3.5.1.32]”, “N-acetylcysteine deacetylase [EC:3.5.1.-]”, “isovaleryl-CoA dehydrogenase [EC:1.3.8.4]”, and “ATP-dependent Clp protease ATP-binding subunit ClpE” over time, while “DNA repair protein RadC” and “pyruvate formate lyase activating enzyme [EC:1.97.1.4]” were increased. Similarly, for the intervention group, “putative DNA primase/helicase”, “UDP-galactopyranose mutase [EC:5.4.99.9]”, “alanyl-tRNA synthetase [EC:6.1.1.7]”, “phage terminase small subunit”, “biofilm protein TabA”, “pyruvyl transferase EpsO [EC:2.-.-.-]”, “CRISPR system Cascade subunit CasB”, and “CRISPR system Cascade subunit CasE” were decreased over the 6-month period, while “pyruvate dehydrogenase (quinone) [EC:1.2.5.1]” and “ATP-dependent RNA helicase CshB [EC:3.6.4.13]” increased.

## 4. Discussion

The relationship between probiotic supplementation and glycemic control in T2D is quite complex and has not been adequately investigated, especially in longer term studies of at least 6 months duration and with HbA1c as the primary endpoint. Most studies have focused on <12 weeks’ evaluation so far, using different strains of probiotics, in different countries, and focusing more on fasting blood glucose, which probably does not allow sufficient time for an effect to be seen and does not give a good index of general glycemic control. Furthermore, the relationship between probiotic administration and the gut microbiome has not been consistently explored. This gap in research may explain the various results that have been found in the literature thus far [48,49]. Furthermore, never has such a study been conducted in Greece. This gap was addressed in the present study by conducting a six-month trial of a proprietary multi-strain probiotic regimen (LactoLevure^R^, comprised of *Lactobacillus acidophilus*, *Lactobacillus plantarum*, *Bifidobacterium* lactis, and *Saccharomyces boulardii*) in individuals with T2D in Greece. The results showed that the probiotic supplementation was well-tolerated and led to positive effects on glycemic and lipid parameters, in addition to improvements in measures of adiposity. While no significant changes were observed in the alpha and beta diversity of the examined gut microbiome in a subgroup of participants, various genera, metabolites, and key enzymes associated with diabetes were influenced by the six-month probiotic intervention. 

The most significant finding involved the effects of probiotic supplementation on glycemia. The analysis of HbA1c values (the primary endpoint of the study), adjusted for changes in waist circumference, revealed compelling insights (Table 3 and Figure 2C). In the control group, there was a slight reduction in mean HbA1c values at both 3 and 6 months (−0.12% and −0.14%, respectively), likely explained by modest decreases in waist circumference (0.41 cm and 0.44 cm, respectively). Conversely, the intervention group displayed more significant reductions in HbA1c levels at both time points (−0.42% and −0.73%, respectively), which were notably different from the control group, even after accounting for a more substantial decrease in waist circumference (3.63 cm at 6 months). These findings underscore the consistent and enduring effectiveness of the probiotic intervention in reducing HbA1c levels, even when considering potential confounding factors like changes in waist circumference. These statistically significant improvements in glycemic control and waist circumference values can bring about clinically meaningful benefits for people with diabetes, including reduced risk of diabetes-related complications (such as cardiovascular disease, neuropathy, retinopathy, and kidney disease), leading to an enhanced quality of life. Moreover, a decrease in waist circumference typically reflects a reduction in visceral fat, which is linked to insulin resistance, and thus, waist circumference reduction can lead to improved insulin sensitivity, making it easier for the body to utilize insulin effectively. This may also allow individuals to reduce their reliance on diabetes medications, which can result in fewer medication side effects and a lower financial burden on healthcare costs. Similar favorable trends were observed in fasting blood glucose (FBG) levels, favoring the intervention group. Notably, individuals with higher baseline glycemia (HbA1c or FBG) experienced more pronounced reductions in glycemic levels with probiotics, a phenomenon commonly seen with other antidiabetic treatments as well [50]. 

The glycemic findings of the present study align with recent umbrella meta-analyses investigating the impact of probiotic supplementation on glycemic control [49,51]. These meta-analyses revealed a significant reduction in mean HbA1c levels associated with probiotic supplementation, with effect sizes of −0.186 (*p* < 0.001) and −0.32% (*p* < 0.001) in the first and second analyses, respectively. However, it is important to note that there was considerable heterogeneity among the studies (*I*^2^ = 72.1%, *p* < 0.001). Similar beneficial effects of probiotics were observed for FBG in these meta-analyses, with effect sizes of −0.408 (*p* < 0.001) and −0.51 mg/dL (*p* < 0.001), respectively, along with substantial heterogeneity (*I*^2^ = 88.1%, *p* < 0.001).

The findings of the favorable impact of probiotics on total cholesterol in the present study are consistent with the broader literature, although exceptions exist [52]. Generally, probiotics have been associated with decreases in total cholesterol and triglycerides, increases in HDL-cholesterol, and no significant effect on LDL-cholesterol [38,48,53]. The mechanisms behind the hypocholesterolemic effect of probiotics encompass various processes, including bile salt deconjugation, modulation of lipid metabolism, reduced intestinal cholesterol absorption through co-precipitation with deconjugated bile salts, incorporation of cholesterol into probiotic cell membranes, conversion of cholesterol into coprostanol in the intestines, and inhibition of the expression of the intestinal cholesterol transporter Niemann–Pick C1 like 1 (NPC1L1) in enterocytes [54].

Adiposity measures were also improved with probiotic treatment in the present study, corroborating results in the literature that probiotics can have beneficial effects on obesity, both in healthy subjects [53,55] as well as in persons with T2D [56]. 

The potential mechanisms by which probiotic supplements may exert beneficial effects in people with diabetes are complex and not fully understood. However, several hypotheses and mechanisms have been proposed. Probiotics are live microorganisms that, when consumed, can influence the composition and balance of the gut microbiota [38]. A healthy gut microbiome is essential for various metabolic functions, including the regulation of blood glucose levels. Probiotic bacteria, particularly certain strains of *Bifidobacteria* and *Lactobacilli*, can ferment dietary fiber in the colon, producing short-chain fatty acids (SCFAs) such as acetate, propionate, and butyrate. SCFAs have been shown to improve insulin sensitivity, enhance glucose metabolism, and reduce inflammation [57]. Chronic inflammation is associated with insulin resistance and diabetes complications. Probiotics may have anti-inflammatory properties and help reduce systemic inflammation, which can contribute to better glycemic control. Some probiotics are thought to enhance the integrity of the intestinal barrier, preventing the leakage of harmful substances from the gut into the bloodstream, like lipopolysaccharides (LPS) [58]. This can reduce low-grade inflammation and improve overall metabolic health. Probiotic bacteria can produce bioactive compounds, such as peptides and neurotransmitters, that may influence gut–brain communication and insulin sensitivity [59]. They may also affect the release of appetite-regulating hormones, such as leptin and ghrelin, potentially helping with weight management [60]. Some probiotic strains have been shown to increase the expression of glucose transporters (e.g., GLUT4) in muscle and adipose tissue, which can enhance glucose uptake and utilization [61]. Furthermore, probiotics may influence bile acid metabolism in the gut, which can have downstream effects on lipid and glucose metabolism [62], and certain probiotics can produce insulin-like peptides that mimic the effects of insulin, potentially improving glucose uptake by cells [63].

Previous studies have recorded many changes in the gut microbiota of T2D individuals, as analyses in gut microbial composition have shown that patients with T2D are characterized by a moderate degree of gut microbial dysbiosis, a reduction in the abundance of some bacteria and an increase in various pathogens [64]. Since the mechanism of the effect of probiotics on glucose control may be the result of changes in microbiota composition, alterations in the gut flora were explored in a subgroup of participants in the present study, through 16S rRNA sequencing. The analysis of α diversity indicated that, while changes occurred over time in the control group, the intervention group demonstrated greater stability in biodiversity. Additionally, β diversity metrics revealed that the qualitative microbial composition remained relatively stable and did not undergo significant changes. These findings align with previous studies suggesting that probiotic consumption may not lead to sustained alterations in gut microbiota diversity and abundance [25,65]. Nevertheless, the transit of bacteria through the gut may still confer certain benefits on glycemic control [48]. 

Although no statistically significant differences were observed in α and β diversity of the examined gut microbiome, several genera, metabolites, and key enzymes associated with diabetes were found to be influenced by the six-month administration of probiotics. Furthermore, it is worth noting that obesity has been proven to be a complex factor influencing the microbiome [66]. Obese individuals, with or without T2D, have different gut microbial compositions, such as a decrease in the genera of *Ruminococcus* and *Akkermansia* [67] or *Haemophilus* [68], but in other studies, an abundance in Lachnospira, Megamonas, and *Haemophilus* has been shown [69]. Most participants in the present study were obese (mean BMI 32.71 kg/m^2^), which may have influenced their gut microbial composition. 

The Tax4Fun2 analysis conducted on the placebo and intervention groups yielded intriguing findings with implications for diabetes. The microbial functional analysis revealed significant dysregulation in several key enzymes. Notably, substantial disparities were observed between the placebo and intervention groups, indicating a behavioral and metabolic shift in the microbiota to adapt to their microenvironment and ensure survival. The placebo group exhibited an augmented presence of microbial metabolites associated with survival, such as the GntR family transcriptional regulator [70], implying a potentially more hostile environment, likely characterized by higher glucose levels, as suggested by the study findings. Interestingly, the distinct profiles of the placebo group were marked by a more aggressive insulin-modulating secondary metabolism. Clp proteins, for instance, play a role in regulating glucose metabolism by activating glucose transporters. Reduced Clp function has been linked to increased fasting glucose and insulin levels, potentially indicating a connection between mitochondrial dysfunction and diabetes [71]. Further scrutiny of the results revealed that microbial metabolism aimed to complement or assist the host, either by catalyzing insulin-modulating mechanisms or targeting glucose production processes. For instance, CshB, an RNA helicase of the DEAD-box protein family, was increased in the intervention group. These proteins can promote translation actions of insulin mRNA and regulate β-cell function and insulin secretion [72]. Other proteins in the same family, like RBPs, are involved in various processes in pancreatic β-cells, including insulin synthesis and secretion. Their expression in glucose-induced β-cells is mediated by the insulin receptor signaling pathway, which can decrease their expression [73].

One notable strength in the present study was the clear documentation of medication use. This was crucial because certain anti-diabetic medications, such as metformin [74] and GLP-1 RAs [75], have been shown to interact with and partially exert their actions through the intestinal microbiome. Since medication type could be a possible confounder related to the association between probiotic use with HbA1c change [48], it was ensured in the present study that medication types and doses remained consistent during the six-month intervention. Of note also, the distribution of these medications was similar between the two groups.

The duration of probiotic intervention can significantly impact glycemic control, with longer interventions often yielding better results [51,76], although this effect is not universally consistent [38]. It is likely that studies of shorter durations (less than three months) may not demonstrate a significant beneficial effect. Additionally, some studies have focused solely on fasting blood glucose instead of HbA1c as an outcome measure, even though HbA1c provides a more comprehensive assessment of overall glycemia [48]. The present study is distinctive in several ways. It is the first in the literature to examine the effects of a multi-strain probiotics formulation on glycemic control in individuals with T2D over a six-month period, with HbA1c as the primary endpoint, involving a Greek population. This is a crucial consideration given the geographical variations in gut microbiome composition influenced by regional dietary and lifestyle factors [36,77].

The strengths of the present study are its relatively long-term follow-up, its random, double-blind distribution of participants with stable lifestyle/dietary and medical treatment over the course of the study, and the fact that stool microbial analysis was performed, aiming to identify differences in microbial composition mediated by the disease and the intervention. 

However, this study had some limitations as well. The participant sample size, particularly in the microbiome sub-study, was not large, and there was a relatively high dropout rate of 32.6%, primarily due to withdrawal of consent. This issue is not unique to this study, as other probiotics trials have also encountered similar dropout rates [24,25]. Additionally, probiotic supplements are not standardized, and their efficacy can vary widely between different products and strains. Therefore, the results of the present study may not be generalizable to all probiotics available on the market. The effectiveness of probiotics for glycemic control is known to be influenced by specific strains, dosages, and individual factors, and some individuals may experience side effects such as bloating, gas, or diarrhea. Lastly, although the quality-of-life questionnaire used in the present study was not standardized, it indicated that the intervention was well-tolerated and had no adverse effects.

## 5. Conclusions

In summary, the present study demonstrated that a six-month administration of the multi-strain probiotic LactoLevure^R^ (containing *Lactobacillus acidophilus*, *Lactobacillus plantarum*, *Bifidobacterium lactis*, and *Saccharomyces boulardii*) among individuals with T2D was well-tolerated and yielded positive effects on both glycemic and lipid parameters, alongside improvements in measures of adiposity. While the investigation did not reveal statistically significant differences in the α- and β diversity of the examined gut microbiome within a subgroup of participants, it did uncover noteworthy impacts on specific genera, metabolites, and key enzymes associated with diabetes due to the six-month probiotic intervention.

It is worth noting that, in the broader context, probiotics offer promise for enhancing glycemic control and overall health in individuals with diabetes mellitus. However, it is essential to carefully consider the pros and cons, particularly regarding the choice of probiotic strains and dosages. Before initiating any probiotic supplement regimen, individuals with diabetes should seek guidance from their healthcare provider to ensure its suitability for their unique circumstances and to monitor its effects attentively. Furthermore, maintaining a balanced diet and regular exercise regimen remains integral to diabetes management. Additionally, it is crucial to acknowledge that high-quality probiotic supplements can be costly, and the long-term financial implications should be taken into account. Further research is warranted to establish definitive recommendations in this domain.

## Figures and Tables

**Table 1:** Baseline demographic, clinical, and laboratory characteristics of the intervention and control groups (mean (SD)).

	Total (n = 91)	Intervention (n = 46)	Control (n = 45)	*p* *
Males/ Females [n (%)]	57/34 (62.6/37.4)	30/16 (65.2/34.8)	27/18 (60/40)	NS
Age (years)	65.12 (10.92)	64.54 (11.12)	65.71 (10.82)	NS
Weight (kg)	92.14 (21.12)	91.39 (21.7)	92.92 (20.72)	NS
BMI (kg/m^2^)	32.71 (6.91)	32.11 (6.55)	33.32 (7.28)	NS
WC (cm)	110.11 (14.57)	108.67 (14.6)	111.58 (14.56)	NS
SBP (mmHg)	130.02 (13.13)	128.26 (13.59)	131.82 (12.54)	NS
DBP (mmHg)	79.49 (10.72)	79.26 (11.87)	79.73 (9.52)	NS
FBG (mmol/L)	7.62 (1.32)	7.54 (1.22)	7.69 (1.43)	NS
HbA1c (%)	7.28 (0.50)	7.24 (0.49)	7.32 (0.52)	NS
TC (mmol/L)	3.94 (0.77)	3.88 (0.76)	4.01 (0.79)	NS
Trig (mmol/L)	1.47 (0.47)	1.44 (0.51)	1.51 (0.43)	NS
HDL-C (mmol/L)	1.13 (0.29)	1.14 (0.30)	1.11 (0.28)	NS
LDL-C (mmol/L)	2.16 (0.67)	2.08 (0.57)	2.25 (0.76)	NS
Creatin (umol/L)	80.46 (23.87)	84.88 (26.53)	76.93 (19.45)	NS
AST (U/L)	19.95 (6.95)	20.30 (5.61)	19.58 (8.14)	NS
ALT (U/L)	21.42 (9.67)	21.41 (6.92)	21.43 (11.93)	NS
γGT (U/L)	21.60 (11.58)	20.69 (10.63)	22.53 (12.53)	NS
Alk Phos (U/L)	65.85 (24.28)	64.60 (18.67)	67.13 (29.44)	NS

* Comparison between intervention and control groups; NS = not significant. BMI: body mass index; WC: waist circumference; SBP: systolic blood pressure; DBP: diastolic blood pressure; FBG: fasting blood glucose; HbA1c: glycated hemoglobin; TC: total cholesterol; Trig: triglycerides, HDL-C: high density lipoprotein cholesterol; LDL-C: low density lipoprotein cholesterol; Creatin: creatinine; AST: aspartate aminotransferase; ALT: alanine aminotransferase; γGT: γ-glutamyl-transferase; Alk Phos: alkaline phosphatase.

**Table 2:** Anthropometric measures before and after intervention with the probiotic or placebo (mean (SD)).

	Probiotics (n = 46)			Placebo (n = 45)			*p* *
	Baseline	3 Months	6 Months	Baseline	3 Months	6 Months	
**BMI (kg/m^2^)**	32.11 (6.55)	31.59 (6.36)	31.27 (6.31)	33.32 (7.28)	33.17 (7.07)	33.16 (6.95)	NS
Change at 3 months		−0.52 (0.63)			−0.15 (1.65)		
Change at 6 months			−0.84 (1.05)			−0.16 (2.34)	
**WC** **(cm)**	108.67 (14.60)	106.85 (14.34)	105.04 (14.13)	111.58 (14.56)	111.17 (13.86)	111.13 (13.52)	**<0.001**
Change at 3 months		−1.83 (1.96)			−0.41 (3.25)		
Change at 6 months			−3.63 (3.10)			−0.44 (5.44)	
**SBP (mmHg)**	128.26 (13.59)	127.93 (12.02)	127.30 (8.48)	131.82 (12.54)	131.02 (14.02)	129.71 (16.10)	NS
Change at 3 months		−0.33 (10.32)			−0.80 (7.84)		
Change at 6 months			−0.96 (13.32)			−2.11 (12.53)	
**DBP (mmHg)**	79.26 (11.87)	78.28 (11.51)	78.85 (12.66)	79.73 (9.52)	78.49 (9.90)	77.96 (10.52)	NS
Change at 3 months		−0.98 (5.16)			−1.24 (4.86)		
Change at 6 months			−0.41 (9.5)			−1.78 (6.59)	

* *p*: comparison between groups by repeated-measure ANOVA. BMI: body mass index; WC: waist circumference; SBP: systolic blood pressure; DBP: diastolic blood pressure.

**Table 3:** Changes in glycemic and lipid parameters before and after intervention with the probiotic or placebo (mean (SD)).

	Probiotics (n = 46)			Placebo (n = 45)			*P*
	Baseline	3 Months	6 Months	Baseline	3 Months	6 Months	
**HbA1c (%)**	7.24 (0.49)	6.82 (0.39)	6.51 (0.44)	7.32 (0.52)	7.19 (0.42)	7.18 (0.45)	**<0.001**
Change at 3 months		−0.42 (0.27)			−0.12 (0.32)		
Change at 6 months			−0.73 (0.42)			−0.14 (0.46)	
**FBG (mmol/L)**	7.55 (1.22)	6.70 (0.85)	6.16 (0.83)	7.70 (1.42)	7.35 (1.13)	7.42 (1.42)	**<0.001**
Change at 3 months		−0.85 (0.95)			−0.35 (0.88)		
Change at 6 months			−1.39 (1.08)			−0.28 (0.96)	
**TC (mmol/L)**	3.87 (0.76)	3.71 (0.69)	3.59 (0.65)	4.00 (0.79)	3.95 (0.80)	4.01 (0.82)	**0.012**
Change at 3 months		−0.16 (0.19)			−0.05 (0.42)		
Change at 6 months			−0.28 (0.27)			0.01 (0.50)	
**Trig (mmol/L)**	1.43 (0.51)	1.37 (0.46)	1.34 (0.46)	1.51 (0.43)	1.51 (0.45)	1.51 (0.46)	NS
Change at 3 months		−0.07 (0.09)			−0.01 (0.19)		
Change at 6 months			−0.09 (0.13)			0.00 (0.25)	
**HDL-C (mg/dL)**	1.13 (0.30)	1.15 (0.29)	1.16 (0.28)	1.11 (0.28)	1.10 (0.27)	1.10 (0.28)	NS
Change at 3 months		0.01 (0.04)			−0.01 (0.08)		
Change at 6 months			0.02 (0.07)			−0.02 (0.13)	
**LDL-C (mg/dL)**	2.08 (0.57)	2.01 (0.56)	2.00 (0.56)	2.24 (0.75)	2.18 (0.73)	2.22 (0.71)	NS
Change at 3 months		−0.07 (0.13)			−0.07 (0.28)		
Change at 6 months			−0.08 (0.19)			−0.03 (0.30)	

*P*: Comparison between groups by the two-factor mixed repeated-measure ANOVA adjusted for change in WC at 6 months. HbA1c: glycated hemoglobin; FBG: fasting blood glucose; TC: total cholesterol; Trig: triglycerides, HDL-C: high density lipoprotein cholesterol; LDL-C: low density lipoprotein cholesterol.

## Data Availability

Raw data for this study have been deposited in the National and Kapodistrian University of Athens “Pergamos” Repository, and can be found at: https://pergamos.lib.uoa.gr/uoa/dl/object/3356123 (accessed on 22 September 2023).

## Supplementary Materials

The following supporting information can be downloaded at: https://www.mdpi.com/article/10.3390/nu15214663/s1, Table S1: Baseline demographic, clinical, and laboratory characteristics of the intervention and control groups participating in the microbiome sub-study (mean (SD)). Table S2. Anthropometric measures before and after intervention with probiotics or placebo in the microbiome sub-study (mean (SD)). Table S3. Changes in glycemic and lipid parameters before and after the intervention with probiotics or placebo in the microbiome sub-study (mean (SD)). Figure S1. Flowchart detailing participants’ recruitment, randomization, and allocation for the placebo and intervention in the microbiome sub-group.
