# Supplementary material for: The Effect of Probiotic Supplements on Metabolic Parameters of People with Type 2 Diabetes in Greece—A Randomized, Double-Blind, Placebo-Controlled Study
Source: Nutrients. 2023 Nov 3;15(21):4663. doi: 10.3390/nu15214663 (PMC10647535; doi:10.3390/nu15214663)
Supplement: Supplementary file 1 [file nutrients-15-04663-s001.zip › nutrients-2652354-supplementary.pdf]

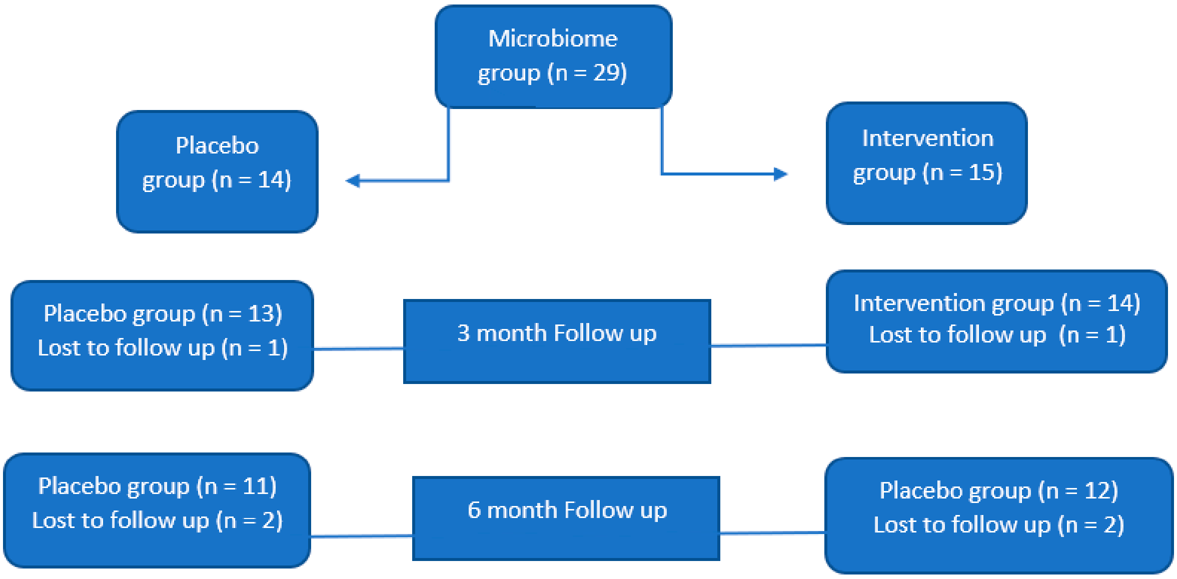

Figure S1. Flowchart detailing participants' recruitment, randomization, and allocation for the placebo and intervention in the microbiome sub-group.

Table S1: Baseline demographic, clinical, and laboratory characteristics of the intervention and control groups participating in the microbiome sub-study [mean (SD)].

	**Total (n=23)**	**Intervention (n=12)**	**Control (n=11)**	***p****
Males/ Females [n (%)]	13 (56.5) / 10 (43.5)	7 (58.3%) / 5 (41.7)	6 (54.5%) / 5 (45.5)	NS
Age (years)	64.09 (12.47)	60.58 (12.55)	67.91 (11.75)	NS
Weight (kg)	96.34 (19.54)	90.46 (14.13)	102.75 (23.11)	NS
BMI (kg/m^2)	34.56 (7.64)	32.43 (5.9)	36.89 (8.87)	NS
WC (cm)	111.87 (12.65)	104.42 (9.86)	120.00 (10.3)	**0.001**
SBP (mmHg)	129.43 (12.97)	124.83 (15.11)	134.45 (8.13)	NS
DBP (mmHg)	78.74 (11.32)	80.42 (11.48)	76.91 (11.39)	NS
FBG (mmol/L)	7.40 (0.91)	7.68 (1.14)	7.11 (0.44)	NS
HbA1c (%)	7.31 (0.50)	7.45 (0.58)	7.16 (0.38)	NS
TC (mmol/L)	3.78 (0.67)	3.75 (0.80)	3.81 (0.54)	NS
Trig (mmol/L)	1.48 (0.45)	1.55 (0.47)	1.40 (0.43)	NS
HDL-C (mmol/L)	1.03 (0.23)	1.09 (0.24)	0.96 (0.20)	NS
LDL-C (mmol/L)	2.07 (0.48)	2.13 (0.34)	2.01 (0.60)	NS
Creatin (umol/L)	79.58 (30.06)	86.65 (37.14)	60.13 (16.8)	NS
AST (U/L)	20.39 (0.56)	20.58 (5.73)	20.18 (9.46)	NS
ALT (U/L)	20.65 (9.97)	20.83 (6.86)	20.46 (12.92)	NS
γGT (U/L)	18.74 (7.68)	19.33 (6.73)	18.09 (8.88)	NS
Alk Phos (U/L)	60.30 (14.84)	55.83 (16.85)	65.18 (11.03)	NS

*Comparison between intervention and control groups; NS = not significant. BMI: body mass index; WC: waist circumference; SBP: systolic blood pressure; DBP: diastolic blood pressure; FBG: fasting blood glucose; HbA1c: glycated hemoglobin; TC: total cholesterol; Trig: triglycerides, HDL-C: high density lipoprotein cholesterol; LDL-C: low density lipoprotein cholesterol; Creatin: creatinine; AST: aspartate aminotransferase; ALT: alanine aminotransferase; γGT: γ-glutamyl-transferase; Alk Phos: alkaline phosphatase.

Table S2. Anthropometric measures before and after intervention with probiotics or placebo in the microbiome sub-study [mean (SD)].

	**Probiotics (n=12)**			**Placebo (n=11)**			***P*** *
	Baseline	3 mos	6 mos	Baseline	3 mos	6 mos	
**BMI (kg/m²)**	32.43 (5.9)	31.87 (5.51)	31.49 (5.67)	36.89 (8.87)	37.10 (8.90)	37.57 (8,90)	**0.017**
Change at 3 mos		-0.56 (0.88)			0.21 (0.75)		
Change at 6 mos			-0.94 (1.56)			0.68 (1.44)	
**WC (cm)**	104.42 (9.86)	103.00 (9.29)	101.17 (8.85)	120.00 (10.30)	121.09 (10.52)	122.18 (10.63)	**0.001**
Change at 3 mos		-1.42 (1.51)			1.09 (2.21)		
Change at 6 mos			-3.25 (2.77)			2.18 (4.26)	
**SBP (mmHg)**	124.83 (15.11)	123.33 (9.24)	127.33 (5.93)	134.45 (8.13)	134.45 (10.01)	135.36 (14.75)	NS
Change at 3 mos		-1.50 (11.74)			0.00 (7.21)		
Change at 6 mos			2.50 (17.78)			0.91 (13.25)	
**DBP (mmHg)**	80.42 (11.48)	80.00 (10.50)	78.55 (15.12)	76.91 (11.39)	78.82 (12.68)	78.55 (15.08)	NS
Change at 3 mos		-0.42 (2.57)			1.91 (3.86)		
Change at 6 mos			2.33 (5.91)			1.64 (7.76)	

**P*: comparison between groups by repeated measures ANOVA. BMI: body mass index; WC: waist circumference; SBP: systolic blood pressure; DBP: diastolic blood pressure

Table S3. Changes in glycemic and lipid parameters before and after the intervention with probiotics or placebo in the microbiome sub-study [mean (SD)].

	**Probiotics (n=46)**			**Placebo (n=45)**			***P***
	Baseline	3 mos	6 mos	Baseline	3 mos	6 mos	
**HbA1c (%)**	7.45 (0.58)	6.98 (0.38)	6.73 (0.47)	7.16 (0.38)	7.20 (0.44)	7.18 (0.52)	**0.026**
Change at 3 mos		-0.48 (0.30)			0.04 (0.12)		
Change at 6 mos			-0.73 (0.48)			0.02 (0.28)	
**FBG (mmol/L)**	7.68 (1.15)	6.59 (0.66)	6.17 (0.70)	7.11 (0.44)	6.99 (0.43)	7.15 (0.67)	**0.057**
Change at 3 mos		-1.09 (0.83)			-0.12 (0.31)		
Change at 6 mos			-1.51 (1.16)			0.04 (0.61)	
**TC (mmol/L)**	3.75 (0.80)	3.63 (0.71)	3.54 (0.65)	3.82 (0.54)	3.79 (0.47)	3.97 (0.57)	NS
Change at 3 mos		-0.12 (0.26)			-0.02 (0.19)		
Change at 6 mos			-0.21 (0.35)			0.16 (0.34)	
**Trig (mmol/L)**	1.55 (0.47)	1.49 (0.43)	1.47 (0.46)	1.40 (0.43)	1.48 (0.47)	1.60 (0.53)	**0.030**
Change at 3 mos		-0.06 (0.07)			0.08 (0.08)		
Change at 6 mos			-0.07 (0.11)			0.20 (0.15)	
**HDL-C (mmol/L)**	1.09 (0.24)	1.10 (0.26)	1.12 (0.27)	0.96 (0.20)	0.93 (0.17)	0.91 (0.16)	NS
Change at 3 mos		0.01 (0.04)			-0.03 (0.06)		
Change at 6 mos			0.03 (0.07)			-0.05 (0.12)	
**LDL-C (mmol/L)**	2.13 (0.34)	2.05 (0.34)	2.10 (0.40)	2.01 (0.60)	1.97 (0.52)	2.02 (0.53)	NS
Change at 3 mos		-0.09 (0.13)			-0.04 (0.12		
Change at 6 mos			-0.03 (0.26)			0.01 (0.10)	

P: comparison between groups by the two-factor mixed repeated measures ANOVA, adjusted for change in WC at 6 months. HbA1c: glycated hemoglobin; FBG: fasting blood glucose; TC: total cholesterol; Trig: triglycerides, HDL-C: high density lipoprotein cholesterol; LDL-C: low density lipoprotein cholesterol
